# Beyond Detection Limits: Integrated Biosensors for Molecular Diagnostics, Longitudinal Monitoring, and Clinical Translation

**DOI:** 10.3390/bios16090477

**Published:** 2026-08-31

**Authors:** Nilanjan Roy, Michael Powers, Minelly Gonzalez, Luca Cucullo

**Affiliations:** 1 Department of Computer Sciences, University of Wisconsin-Madison, 1210 West Dayton Street, Madison, WI 53706, USA; nroy8@wisc.edu; 2 Department of Biological and Biomedical Sciences, Oakland University, Rochester, MI 48309, USA; michaelpowers@oakland.edu (M.P.); mkgonzalez@oakland.edu (M.G.); 3 Department of Foundational Medical Studies, Oakland University William Beaumont School of Medicine, Rochester, MI 48309, USA

**Keywords:** biointerface engineering, continuous molecular monitoring, wearable biosensors, microneedle biosensors, CRISPR diagnostics, molecular diagnostics, clinical validation, clinical translation

## Abstract

Biosensors are evolving from isolated analytical detectors into integrated systems for molecular diagnosis, longitudinal monitoring, dynamic tissue assessment, and clinical or preclinical decision support. This critical narrative review synthesizes literature from 2018 through July 2026 on biorecognition, biointerface engineering, transduction, wearable and microneedle architectures, CRISPR diagnostics, and sensor-integrated microphysiological systems. Unlike previous work that primarily classifies biosensors by analyte, recognition chemistry, or transduction modality, this review provides a unified translational framework that shifts evaluation from analytical sensitivity alone to the ability of systems to generate reliable, longitudinal, and clinically actionable information. A central distinction is made among snapshot assays, repeated discrete measurements, and genuine molecular trajectories, which require reversible recognition, controlled sampling, calibration, drift management, and temporal fidelity. Across applications, translational maturity depends on the measurement pathway. Continuous glucose monitoring remains the clearest benchmark because it combines durable chemistry, reproducible manufacture, workflow integration, and demonstrated clinical benefit. Other platforms have reached authentic-matrix testing, feasibility, prospective validation, or regulatory clearance but remain limited by fouling, matrix effects, calibration transfer, incomplete sample-to-answer operation, device variability, and insufficient manufacturing evidence. Future progress requires durable interfaces, claim-matched validation, reproducible scale-up, interoperable data systems, and measurements that remain trustworthy across time, users, devices, and settings.

## 1. Introduction

Biosensors convert a biological recognition event into a measurable signal. Historically, this definition encompassed devices ranging from enzyme electrodes to immunoassays and optical affinity sensors. The field is now undergoing a broader transition. Biosensors are increasingly expected not only to detect a molecular target but also to quantify it in complex biological matrices and operate near the patient. They must also track changes over time, integrate with portable readers and software, and provide information that supports clinical or experimental decisions. This progression has expanded the role of the biosensor from an isolated analytical detector toward a complete measurement system comprising biorecognition chemistry, a biointerface, a transducer, a sampling architecture, calibration and data-processing methods, and an intended-use workflow.

Within this review, the term biosensor is used broadly to encompass integrated molecular measurement systems in which biological recognition is coupled to a transduction mechanism and a defined diagnostic, monitoring, or experimental output. Accordingly, this review encompasses both single-use or disposable biosensors that provide diagnostic snapshots and wearable, implantable, and other longitudinal systems designed for repeated or continuous monitoring, as well as point-of-care molecular diagnostics, instrumented laboratory platforms, and sensor-integrated microphysiological systems. The performance requirements applied to each platform are matched to its intended measurement class and use. Biomarker-discovery studies and conventional laboratory assays are discussed only when they establish a clinical benchmark, intended-use context, or translational comparator for an integrated biosensing platform.

Two established technologies illustrate the ends of this translational spectrum. Enzyme-coupled electrochemical sensing demonstrated that biochemical concentrations could be measured repeatedly and ultimately enabled continuous glucose monitoring [1]. At the other extreme, digital single-molecule immunoassays showed that extremely low concentrations of proteins could be quantified in serum through molecular counting [2]. These examples represent different forms of analytical success. Continuous glucose monitoring produces a longitudinal trajectory that can guide treatment over days, whereas digital immunoassays provide highly sensitive measurements from discrete samples. Both are valuable, but they answer different clinical questions and impose different requirements on recognition kinetics, sampling, calibration, instrumentation, and validation.

This distinction between a measurement and a trajectory is central to the present review. A snapshot diagnostic estimates an analyte or classifies a condition at one time point. Intermittent testing repeats discrete measurements. Genuine continuous or near-continuous monitoring requires the sensor to respond reversibly as concentration rises and falls, remain calibrated during prolonged exposure to a biological environment, control access to the sampled compartment, and distinguish physiological change from drift, fouling, transport delay, and device motion. Repeated measurements should therefore not automatically be described as continuous monitoring, and a short, controlled trace should not be interpreted as evidence of durable longitudinal operation. Effective temporal resolution is determined not only by nominal sampling frequency but also by fluid transport, compartmental lag, receptor response and recovery, sensor-memory effects, and signal processing.

The same distinction applies to clinical translation. Biosensor performance is frequently summarized by analytical sensitivity or limit of detection, yet these measures capture only one part of the complete system. In authentic samples, receptor affinity interacts with surface orientation, receptor density, nonspecific adsorption, matrix interference, transport, and transducer stability. A device that performs exceptionally in buffer may lose accuracy in serum, whole blood, sweat, interstitial fluid, or tissue. Similarly, strong discrimination in a small retrospective case–control cohort may not persist in a prospective intended-use population, where prevalence, comorbidities, borderline cases, preanalytical variation, and operator error influence predictive performance.

Biosensors must therefore be evaluated according to the information and decision they are intended to support. Infectious-disease testing often prioritizes rapid and accurate pathogen identification together with complete sample-to-answer operation. Cardiometabolic monitoring requires stable longitudinal measurement and, in the most mature applications, integration with treatment. Oncology screening demands high specificity in low-prevalence populations, reliable early-stage detection, and an acceptable pathway for resolving positive results. Neurodegeneration increasingly relies on ultrasensitive blood biomarkers, but clinical value depends on standardized thresholds, confirmatory testing, and integration into diagnostic and therapeutic pathways. Sensor-integrated organ-on-chip systems address a different use case: they monitor dynamic tissue states during disease modeling and drug evaluation rather than diagnosing a patient directly.

Recent advances have expanded every layer of the biosensor system. Aptamers, resettable antibody switches, and engineered affinity reagents have enabled reversible molecular recognition. CRISPR-associated nucleases have introduced programmable nucleic-acid detection. Anti-fouling polymers, hydrogels, nanostructured interfaces, and oriented receptor immobilization have improved operation in complex matrices. Electrochemical, optical, plasmonic, digital, nanophotonic, and field-effect-transistor platforms offer complementary combinations of sensitivity, portability, multiplexing, temporal resolution, and manufacturing complexity. Wearable sweat patches, microneedle arrays, implantable sensors, and microphysiological systems have further shifted attention from isolated detection toward controlled sampling and longitudinal information.

Despite this progress, clinical translation remains uneven. Continuous glucose monitoring has achieved manufacturing scale, workflow integration, regulatory maturity, and demonstrated clinical benefit, but its success has not transferred automatically to other metabolites, proteins, hormones, drugs, or multiplexed panels. CRISPR diagnostics have reached authentic clinical specimens and limited field deployment, yet extraction, amplification, reagent storage, internal controls, and decentralized usability remain limiting. Liquid-biopsy and neurodegeneration assays have produced major advances in molecular detection, but analytical validity, clinical validity, regulatory clearance, and demonstrated improvement in patient outcomes remain distinct stages. Across these domains, the major bottlenecks increasingly involve interface stability, matrix robustness, calibration, sampling reliability, prospective validation, reproducible manufacture, software governance, and implementation rather than another reduction in detection limit.

The distinctive contribution of this review is a cross-cutting framework that evaluates biosensor systems according to the type of information they produce and the strength of evidence supporting their intended use. Rather than organizing platforms solely by analyte, recognition chemistry, or transduction modality, the framework distinguishes diagnostic snapshots, repeated discrete measurements, and genuine molecular trajectories, each of which imposes different requirements for reversibility, sampling, temporal fidelity, calibration, and drift control. It then evaluates the complete measurement chain from biorecognition and biointerface design through transduction, sampling, data interpretation, validation, manufacturing, and workflow integration and assesses translational maturity according to the highest evidence tier directly demonstrated. This structure enables technologies from different application domains to be compared on a common basis while preserving the analytical, temporal, and clinical distinctions that determine whether a biosensor can support a defined decision.

Existing frameworks and reviews address important portions of this translational pathway but generally do so within narrower boundaries. End-to-end wearable-sensor frameworks emphasize the integration of materials, sensing, power, and decision-making components [3], while reviews of real-time molecular monitoring focus primarily on the recognition kinetics, signal stability, and in vivo performance required for continuous sensing [4]. Commercial-translation analyses of electrochemical biosensors emphasize manufacturing scale-up, supply-chain, regulatory, and quality-system requirements [5], whereas established CLSI, FDA, and reporting frameworks provide detailed guidance for analytical and clinical validation [6,7,8,9,10,11,12,13,14,15]. The framework used here differs by applying a common, claim-matched evidence structure across single-use diagnostics, longitudinal monitoring systems, and tissue-facing biosensors. It organizes these heterogeneous technologies according to measurement class, snapshot, repeated discrete measurement, or molecular trajectory, and the highest level of translational evidence directly demonstrated, from analytical proof of concept through clinical or preclinical benefit. Accordingly, each technology and application domain is evaluated using the same three questions: what information does the system produce, what is the highest evidence tier demonstrated, and what principal limitation prevents advancement to the next translational stage.

In this work, we focus primarily on studies published from 2018 onward, while drawing on earlier landmark studies when needed to establish the foundations of current platforms. We do not aim to provide a comprehensive catalog of biomarker-discovery studies, fabrication materials, general wearable physiology, or organ-on-chip engineering unless these areas involve integrated molecular measurement. Sensor-integrated microphysiological systems are included in a limited scope as tissue-facing examples of longitudinal biosensing and preclinical decision support. Although recognition chemistry, transduction technologies, and device engineering have advanced substantially, biosensor progress is often reviewed within separate technical domains. Here, we organize the field by the information biosensors produce and the evidence supporting their clinical or experimental use. By integrating biorecognition, biointerface engineering, transduction, sampling design, longitudinal monitoring, validation, manufacturing, and implementation into a single translational framework, this review provides a systems-level basis for comparing biosensor platforms and identifying key barriers to broad clinical adoption.

The central premise is that the next major advances in biosensing will come not from sensitivity alone, but from systems that remain accurate in the intended matrix and setting, stable over the required use period, reproducible across manufactured units and production lots and, where applicable, deployment sites within the defined intended use, interpretable within the appropriate clinical or experimental context, and integrated into a decision pathway. The field’s most important shift is therefore from detecting molecular signals to generating reliable, actionable information.

### Literature Search and Review Framework

This critical narrative review focused primarily on peer-reviewed literature published from January 2018 through 20 July 2026, supplemented by earlier landmark studies and current standards or regulatory documents where needed to establish scientific and translational foundations.

Structured searches of PubMed, Web of Science, and Scopus were conducted through 20 July 2026 using combinations of terms related to biosensors, molecular diagnostics, antibodies, enzymes, nucleic-acid recognition, aptamers, CRISPR, electrochemical, amperometric, potentiometric, impedimetric, field-effect-transistor (FET) and graphene-FET sensing, optical and plasmonic sensing, single-use and point-of-care sensors, continuous monitoring, wearable sensors, microneedles, organ-on-chip systems, clinical validation, manufacturing, data integration, and regulatory translation. Additional literature was identified through targeted and iterative searches of publisher resources, citation networks, and official regulatory-agency and standards-organization websites.

Priority was given to original studies reporting authentic-matrix performance, reversible or longitudinal measurement, integrated sampling and readout, comparison with accepted reference methods, animal or human evaluation, prospective intended-use validation, device reproducibility, regulatory status, or clinical implementation. Earlier foundational studies were included when they established a recognition mechanism, transduction principle, device architecture, or clinical benchmark required to interpret subsequent developments. Review articles were used to establish broader field context and identify relevant primary studies.

Studies focused exclusively on biomarker discovery, whereas isolated material synthesis, transducer characterization without an integrated biosensing function, general wearable physiology, or organ-on-chip engineering without molecular measurement were outside the defined scope. Buffer-based studies were included when they established an important analytical principle but were not used alone to support claims of clinical validity, longitudinal performance, or translational readiness. Regulatory documents, technical standards, and official agency records were included where directly relevant to validation, approval, quality systems, or implementation.

Translational maturity was assessed qualitatively according to the highest evidence tier demonstrated: (1) analytical proof of concept, (2) intended-matrix performance, (3) authentic-specimen testing or animal monitoring, (4) human feasibility, (5) prospective intended-use validation, (6) regulatory clearance or approval, and (7) demonstrated clinical benefit or implementation. These tiers were used as an interpretive framework rather than a formal scoring system, and evidence was evaluated according to the claim directly supported rather than detection limit alone.

Evidence was synthesized using a structured cross-platform framework that compared technologies according to measurement class, intended use, and the highest demonstrated level of translational evidence. This framework enabled comparison across diverse biosensor systems while preserving the analytical, temporal, and clinical distinctions relevant to each application.

Because this was a critical narrative review rather than a quantitative systematic review, the search was designed to support broad, claim-matched synthesis rather than exhaustive retrieval or PRISMA-level systematicity. The substantial heterogeneity in analytes, matrices, device designs, reference standards, study populations, and reported endpoints precluded a meaningful meta-analysis. The evidence tiers should therefore be interpreted as a structured framework for comparison rather than as a formal quantitative ranking or comprehensive assessment of study quality.

## 2. Biorecognition and Biointerface Engineering

**Figure 1:**
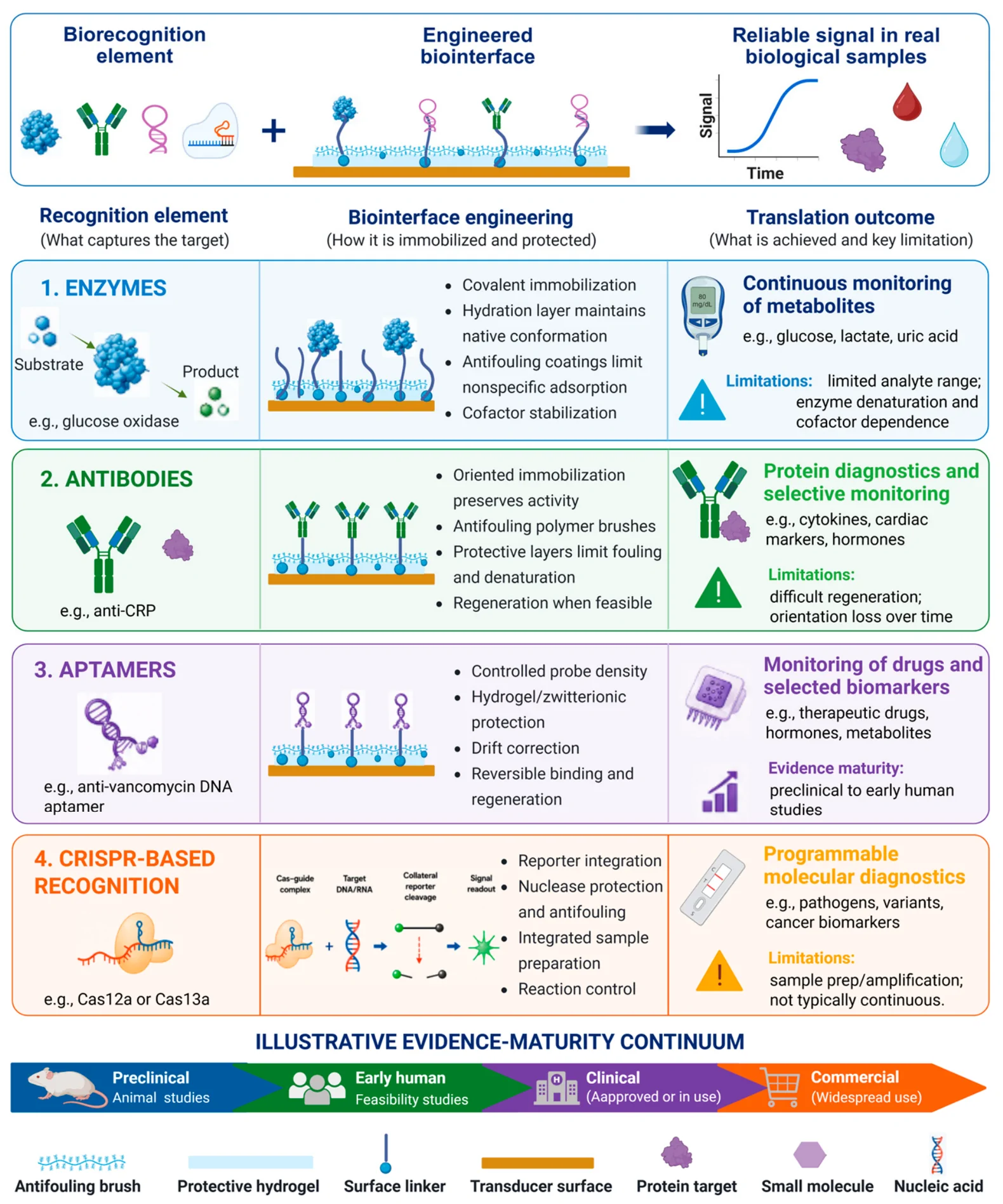
Biorecognition and biointerface engineering for reliable biosensing. Representative recognition elements, biointerface strategies, applications, limitations, and translational maturity are summarized. Created in BioRender. Roy, N. (2026) https://BioRender.com/ikk5xac, accessed on 20 July 2026.

The performance of a biosensor in a biological sample is not determined by receptor affinity alone. It emerges from a coupled system comprising the recognition element, its orientation and density at the transducer, the physicochemical properties of the surrounding interface, and—when repeated measurements are required—the mechanisms for signal reset, calibration, and drift correction. This distinction is central to the field’s evolution from single-use tests toward longitudinal molecular monitoring. Enzymes and antibodies remain indispensable, but aptamers, CRISPR-associated nucleases, resettable affinity switches, anti-fouling materials, and engineered nanostructures have expanded the design space. The resulting lesson is that selectivity in buffer is principally a receptor property, whereas reliable selectivity and stability in blood, serum, sweat, interstitial fluid, or tissue are receptor-plus-interface properties. The principal recognition classes, associated biointerface strategies, applications, limitations, and translational maturity are summarized in Figure 1.

### 2.1. Conventional Receptors and the Emergence of Resettable Protein Recognition

Enzymes established the practical model for continuous biosensing. The Clark oxygen electrode and the subsequent enzyme-electrode concept showed that a catalytic reaction could convert analyte concentration into a continuously renewed electrochemical signal [1]. Glucose sensing succeeded because glucose oxidase combines useful specificity with catalytic turnover, while the electrode and membrane control mass transport, interferents, and access to oxygen or alternative electron acceptors. This architecture remains the translational benchmark: the receptor does not operate independently, but as part of a membrane–enzyme-electrode system engineered for repeatable manufacture and prolonged exposure to biological fluid.

Antibodies provide a different advantage. Their affinity and epitope specificity underpin immunoassays and digital single-molecule protein measurements, including subfemtomolar detection in serum [2]. However, the properties that make high-affinity antibodies effective in endpoint assays can impede continuous monitoring. Slow dissociation causes occupancy to persist after the analyte concentration falls; random immobilization can bury binding sites or create heterogeneous distances from the transducer; and repeated regeneration often requires chemical conditions that damage either the antibody or the surface. Therefore, conventional antibody sensors generally measure accumulated binding rather than an immediately reversible concentration trajectory.

Recent work shows that these constraints can be engineered around, but only by treating resetting and interface protection as primary design functions. Thompson et al. converted existing antibodies into target-responsive molecular switches by coupling antigen recognition to a competitive, fluorescent DNA construct, enabling reversible optical sensing of small molecules without engineering the antibody sequence itself [16]. Zargartalebi et al. addressed the slow off-rate of high-affinity protein recognition through an active-reset strategy: high-frequency electrical oscillations accelerated target dissociation and regenerated the sensor within approximately one minute, permitting repeated in vivo tracking of inflammatory proteins [17]. A complementary approach protected an antibody-based molecular-pendulum sensor inside a zwitterionic microneedle hydrogel, enabling repeated insulin measurements in interstitial fluid in diabetic rats [18]. Collectively, these studies do not show that antibodies are intrinsically suited to continuous monitoring. They show that antibodies become monitor-compatible when molecular competition, active regeneration, and anti-fouling transport layers are deliberately engineered into the sensing system.

### 2.2. Aptamers: From Affinity Reagents to Reversible Molecular Switches

Aptamers are synthetic single-stranded nucleic acids selected to bind molecular targets through sequence-dependent folding. The original SELEX framework established that functional RNA ligands could be enriched from large combinatorial libraries [19]. Their value to biosensing lies not merely in being “synthetic antibodies,” but in their chemical reproducibility, ease of modification, and capacity to undergo binding-induced structural changes. The foundational electrochemical aptamer-based (EAB) architecture placed a redox-labeled aptamer on a gold electrode so that target binding altered electron-transfer kinetics, producing a reagentless signal that could rise and fall with concentration [20]. This reversible format subsequently enabled real-time therapeutic-drug measurements in living animals [21,22]. Aptamer conformational changes were also used in field-effect transistors to reposition charge within the Debye length, permitting label-free small-molecule sensing under physiologically relevant ionic conditions [23].

The major recent advance is a shift from discovering binders to engineering binders for a specified sensing function. Conventional selections often optimize equilibrium affinity under simplified conditions, even though continuous sensors also require appropriate association and dissociation kinetics, structure switching, matrix compatibility, and surface-tethered performance. A high-dimensional microfluidic selection method demonstrated the programmable enrichment of aptamers across defined affinity ranges rather than simply selecting the tightest binders [24]. A non-fouling porous-hydrogel method enabled high-affinity aptamer selection in a single round while suppressing nonspecific interactions [25]. Massively parallel screening has been used to identify sequence variants that convert binding aptamers into signal-generating molecular switches [26]. Single-molecule kinetic analysis further showed that aptamers with similar equilibrium affinity can differ substantially in association, dissociation, and conformational dynamics—properties that directly determine the temporal response of a continuous sensor [27]. These studies redefine aptamer quality: the best monitoring reagent is not necessarily the highest-affinity sequence, but the sequence whose affinity, kinetics, switching amplitude, and stability match the intended concentration range and sampling interval.

Translation into wearable and minimally invasive formats has accelerated since 2022. Microneedle EAB platforms have tracked therapeutic drugs in dermal interstitial fluid, linking reversible recognition to precision-dosing applications [28,29]. Yet these devices also exposed the weakness of the conventional DNA–thiol self-assembled monolayer: serum proteins, elevated temperature, thiol exchange, and nuclease activity progressively degrade the interface. Mechanistic studies of a week-long operation identified monolayer degradation and bio-fouling as separable contributors and showed that surface chemistry, rather than aptamer affinity alone, governs long-duration performance [30]. Hydrogel protection subsequently supported continuous drug measurements at multiple implantation sites in rats [31]. Beyond therapeutic drugs, a strand-displacement aptamer interface integrated with a gold nanoparticle–MXene electrode enabled wearable estradiol measurements in human sweat [32]. Clinical-sample validation is also becoming more rigorous: an EAB vancomycin assay was compared to automated immunoassays using authentic serum samples and revealed an analytically important distinction between the free drug measured by the aptamer sensor and the total drug reported by the reference method [33]. Dual-purpose aptamer architectures have also been designed to combine sensing with internal referencing or multiplexable metabolic monitoring [34].

The strongest recent studies move beyond hours-long feasibility toward sustained operation and human use. Chen et al. developed a biomimetic, hierarchical nano–bio interface containing a bicontinuous nanoporous structure, polymer protection, and aptamer switches; after one week of intravenous implantation in freely moving rats, the sensor retained stable calibration and substantial baseline signal [35]. A 2026 pilot clinical trial tested a microneedle EAB patch in six healthy participants and recorded dermal-interstitial-fluid vancomycin concentrations at five-minute resolution for 24 h, although degradation limited the most reliable analysis primarily to the first 12 h [36]. In parallel, xenonucleic-acid substitution improved nuclease resistance and supported week-long, seconds-resolved in vivo drug measurements [37]. These papers represent a meaningful translational progression—from in vitro reversibility to animal pharmacokinetics, to first-in-human trajectories—but they also show that receptor degradation, insertion-site variability, calibration transfer, and wireless sample-to-answer integration remain unresolved.

A further conceptual advance concerns the affinity–speed trade-off. Low-abundance proteins are often measured using high-affinity binders, but their slow dissociation can prevent rapid tracking as concentrations decrease. Single-molecule readout of reversible nanoswitches offers a different route: rather than measuring ensemble occupancy at equilibrium, it counts association and dissociation events. Modeling and experimental validation indicate that low-affinity, rapidly reversible interactions can, in principle, support low-picomolar quantification on minute timescales when enough individual switching events are resolved [38]. This does not yet constitute a deployable clinical sensor, but it changes the design logic for continuous protein monitoring. High affinity, fast response, and reversibility need not always be optimized in the same molecule; they can be distributed across receptor kinetics, nanoswitch architecture, and readout statistics.

### 2.3. CRISPR-Based Programmable Recognition

CRISPR diagnostics introduced a different form of biorecognition: sequence-programmable target identification coupled to enzymatic signal amplification. The foundational SHERLOCK study showed that target recognition by Cas13 activates the collateral cleavage of nearby RNA reporters [39]. Soon afterward, Cas12a target binding was shown to unleash nonspecific single-stranded DNase activity, providing an analogous route to DNA detection [40]. These mechanisms transformed guide-RNA sequence design into a programmable recognition layer and enabled single-nucleotide discrimination, portable fluorescence or lateral-flow readouts, and rapid retargeting to emerging pathogens.

The COVID-19 pandemic tested whether this programmable chemistry could function in clinically relevant workflows. DETECTR combined preamplification with Cas12-based recognition and lateral-flow readout for SARS-CoV-2 [41]. Amplification-free Cas13 detection coupled to mobile-phone microscopy reduced workflow complexity while preserving direct sequence recognition [42]. Engineered Cas12a variants and guide architectures improved collateral-cleavage efficiency and were evaluated against clinical specimens [43]. Multiplexed microfluidic CRISPR platforms then expanded the concept from single-pathogen detection to parallel respiratory-virus and SARS-CoV-2-variant testing across large clinical specimen sets [44]. Together, these studies established CRISPR as a powerful diagnostic recognition chemistry, but not as an inherently sample-to-answer technology: extraction, amplification, fluid handling, contamination control, and interpretation remain separate engineering problems.

Recent work has expanded CRISPR recognition beyond acute infectious-disease testing. CRISPR-amplified urinary nanosensor barcodes enabled the multiplexed and portable detection of tumor-associated protease activity in preclinical cancer models [45]. Asymmetric CRISPR circuits used competitive guide-RNA design to create cascade amplification for nucleic-acid detection [46]. Split-crRNA architectures improved sensitivity and enabled the multiplexed detection of RNA and DNA targets [47]. These innovations increase programmability and analytical gain, but they also reinforce an important boundary for this review: CRISPR biosensors are generally single-use molecular diagnostics, not reversible monitors. Collateral cleavage consumes reporter molecules, most workflows still require target amplification or carefully controlled reaction volumes, and quantitative output remains less mature than qualitative detection. CRISPR therefore bridges molecular recognition and decentralized diagnostics, whereas aptamer and resettable affinity platforms are currently better suited to tracking continuous concentration trajectories.

### 2.4. Biointerface Engineering in Complex Biological Matrices

A recognition element can be highly selective in buffer and still fail in a patient sample. The interface determines whether the receptor remains accessible, correctly oriented, electrically or optically coupled to the transducer, and protected from nonspecific adsorption. Protein and cellular fouling can block target transport, alter interfacial capacitance, increase electron-transfer distance, and create time-dependent drift. Receptor crowding can suppress aptamer folding or antibody accessibility. Conversely, receptor densities that are too low can reduce signal and increase stochastic variability. For FET devices, receptor length and the position of binding-induced charge relative to the electrical double layer are especially consequential. Interface optimization is therefore not a generic coating step; it is a matrix-, transducer-, receptor-, and time-scale-specific design problem.

Several studies established anti-fouling coatings as active analytical components rather than passive supports. A cross-linked nanocomposite containing protein, reduced graphene oxide, and conductive nanomaterials enabled affinity-based electrochemical measurements in undiluted biological fluids while resisting nonspecific adsorption [48]. A rapid coating method later adapted conductive anti-fouling nanomaterials to multiplexed sensing in whole blood, moving the approach closer to scalable fabrication [49]. For EAB sensors, mechanistic analysis separated blood-induced and temperature-dependent components of signal drift and showed why a single correction algorithm cannot substitute for stable surface chemistry [50]. Studies of sweat electrodes further demonstrated that fouling is not simply a uniform protein film: lipid-rich and spatially heterogeneous deposits contribute materially to signal degradation [51]. The protective strategy must therefore be matched to the actual matrix rather than evaluated only with bovine serum albumin or a single model interferent.

Current interface engineering increasingly combines chemical, structural, and mechanical protection. The biomimetic aptamer interface described above used hierarchical pores to preserve analyte transport while polymer layers limited biological attack [35]. The zwitterionic microneedle system protected an antibody sensor while simultaneously extracting interstitial fluid [18]. Self-confined tetrahedral DNA circuits embedded in soft bioelectronics provided defined probe geometry, reduced bio-fouling, resisted repeated mechanical deformation, and enabled multiplexed wound-protein monitoring in diabetic mice [52]. Oriented antibody coupling through glycan remodeling and DNA linkers has likewise been demonstrated on an anti-fouling polymer, directly addressing the loss of activity and variability caused by random immobilization [53]. These examples show that the interface performs multiple functions at once: molecular sieving, anti-fouling, receptor presentation, mechanical compliance, local signal amplification, and sometimes fluid acquisition.

The remaining translational weakness is reproducibility. Academic reports commonly optimize a small number of hand-fabricated devices and emphasize detection limits, while giving less attention to device-to-device variation, coating thickness, receptor density, shelf stability, sterilization, and lot-to-lot manufacturing yield. Yet these parameters determine whether a receptor–interface combination can become a clinical product. A high-performing interface should therefore be evaluated through at least four evidence layers: nonspecific adsorption and receptor activity after fabrication; analytical performance in authentic complex matrices; stability under the intended mechanical and thermal conditions; and reproducibility across operators, fabrication batches, storage periods, and devices. Without these data, improved sensitivity in a single sensor cannot be interpreted as translational readiness.

Overall, modern biorecognition is best understood as co-design among four elements: the receptor defines molecular identity; the interface preserves access and suppresses matrix interference; the reset or regeneration mechanism enables repeated measurement; and calibration or referencing converts a drifting physical signal into a reliable concentration trajectory. Enzymes remain ideal where catalytic turnover is available, antibodies remain dominant for high-specificity protein recognition, aptamers provide the most mature general platform for reagentless reversible small-molecule monitoring, and CRISPR provides uniquely programmable nucleic-acid diagnostics. The decisive advances since 2018 have occurred where these recognition chemistries were paired with purpose-built interfaces rather than treated as isolated molecular components.

## 3. Transduction Mechanisms and Performance Trade-Offs

Transduction determines how a molecular recognition event is converted into an analytical signal, but its practical consequences extend well beyond the nominal limit of detection. The chosen mechanism governs response time, dynamic range, matrix tolerance, label requirements, multiplexing, calibration, power consumption, instrument complexity, fabrication yield, and the feasibility of repeated or continuous use. The modern field emerged from three foundational lines: enzyme-coupled electrochemistry for continuous glucose measurement [1], optical surface-plasmon sensing of interfacial binding [54], and ion-sensitive field-effect devices that converted surface potential into an electronic signal [55]. Contemporary platforms elaborate these principles through flexible electrodes, microfluidics, single-molecule counting, nanophotonics, and transistor arrays. Accordingly, the relevant question is not which modality is universally most sensitive, but which modality produces sufficiently accurate and stable information within the intended biological matrix and clinical workflow.

### 3.1. Electrochemical Transduction

Electrochemical biosensors remain the most mature route to portable and continuous biochemical measurement because the transducer can be miniaturized, fabricated at low cost, and operated with compact, low-power electronics. Amperometric and voltammetric sensors measure faradaic current generated by enzymatic reactions, redox reporters, or the direct oxidation and reduction in the analyte. Potentiometric sensors measure changes in interfacial potential, typically for ions, whereas impedimetric and conductometric approaches track changes in interfacial charge transfer, capacitance, or solution conductivity. These modes share a common advantage: the signal is produced locally at an electrode and can be integrated readily with printed substrates, microfluidics, microneedles, and wireless electronics.

The trajectory from laboratory electrode to integrated monitoring system is clearest in wearable and minimally invasive platforms. Fully integrated sweat arrays established simultaneous amperometric metabolite sensing and potentiometric electrolyte measurement with on-board calibration and wireless transmission [56]. Microneedle arrays subsequently extended electrochemical monitoring to interstitial fluid, including simultaneous glucose, lactate, and alcohol measurements [57], while regenerable affinity electrodes enabled the repeated monitoring of metabolites and nutrients in sweat [58]. Human translation has also progressed beyond benchtop demonstrations: a first-in-human microneedle study tracked phenoxymethylpenicillin pharmacokinetics and compared sensor measurements to serum and microdialysis data [59], and a regenerable immunoassay device recently supported repeated C-peptide measurements through a minimally invasive wearable format [60]. These studies show that electrochemical transduction is no longer limited to catalytic small-molecule sensing, although evidence for clinical benefit remains much stronger for glucose than for newer targets.

Recent systems increasingly address sampling and actionability rather than sensitivity alone. A wearable electrochemical/biophysical platform measured sweat volume and sodium concentration during demanding field use and coupled the data to haptic feedback, with on-body comparisons against reference measurements [61]. In 2026, an ultrasound-assisted wearable device induced sweat under resting conditions and integrated the electrochemical measurement of uric acid, pH, and potassium, addressing the persistent limitation that many sweat sensors operate only during exercise or pharmacological iontophoresis [62]. Differential microneedle architectures and fully integrated multiplex arrays likewise use reference channels, common-mode rejection, and system-level packaging to stabilize on-body measurements [63,64]. These developments are important because continuous monitoring requires the control of sampling rate, fluid transport, temperature, motion, and reference-electrode behavior in addition to analyte recognition.

The limitations of electrochemical sensing are equally structural. Bio-fouling and mediator loss alter electron transfer; oxygen dependence can bias first-generation oxidase sensors; reference potentials drift; and surface area, enzyme loading, membrane thickness, and mass transport vary across devices. Calibration can therefore dominate performance even when analytical sensitivity is high. The clinical maturity of continuous glucose monitoring reflects decades of progress in membranes, factory calibration, electronics, manufacturing control, and outcome studies, not amperometry alone. A 24-week randomized trial of intermittently scanned glucose monitoring demonstrated improved glycemic outcomes, illustrating the level of clinical evidence that newer electrochemical sensors have not yet approached [65]. For emerging targets, the strongest claims should therefore be limited to the demonstrated tier: analytical feasibility, complex-matrix operation, animal monitoring, short human feasibility, or prospective clinical benefit.

### 3.2. Optical, Plasmonic, Digital, and Nanophotonic Transduction

Optical biosensing encompasses several physically distinct readouts. Fluorescence and colorimetry use labels or reaction products; surface-plasmon resonance measures refractive-index changes at a metal interface; surface-enhanced Raman scattering amplifies molecular vibrational signatures; and photonic resonators, interferometers, and metasurfaces convert binding into wavelength, phase, or intensity changes. Optical methods are particularly strong when spatial multiplexing, visual readout, or extremely low analyte abundance is decisive. They are also less constrained by electrical reference electrodes, although they introduce their own dependencies on illumination stability, optical alignment, background scattering, and detector performance.

The most consequential optical advance has been the conversion of analog intensity into digital molecular counts. Single-molecule arrays partition enzyme-labeled immunocomplexes into femtoliter wells and count positive compartments, enabling subfemtomolar protein detection [2]. Automation and multiplexing transformed this concept into an instrumented laboratory platform [66]. Subsequent systems shortened assay time or increased multiplexing: pre-equilibrium digital ELISA captured early sandwich-complex formation for the rapid serial measurement of inflammatory proteins [67]; high-throughput on-bead amplification expanded multiplexed attomolar detection [68]; and DigitISA performed surface-free, solution-phase digital protein counting using microfluidic separation and single-molecule detection [69]. These approaches substantially improve analytical sensitivity, but repeated measurements of sequential blood samples should not be described as continuous in vivo sensing. They remain discrete assays whose temporal resolution is limited by sampling and workflow.

Plasmonic and nanophotonic platforms occupy a broad middle ground between visual point-of-care tests and centralized digital instruments. A dual-functional plasmonic photothermal sensor used localized heating and refractive-index sensing to improve SARS-CoV-2 sequence discrimination [70], while portable nanoplasmonic imaging enabled the rapid digital detection of inflammatory biomarkers [71]. More recently, a nanophotonic S100B platform combined optical amplification with deep-learning quantification in a time-controlled neurosurgical cohort [72]. This study illustrates both the opportunity and the caution: nanophotonic readout can resolve low-abundance, rapidly changing biomarkers, but algorithm-assisted performance in a narrow clinical model does not establish general diagnostic utility.

Continuous optical affinity sensing remains difficult because high-affinity binding produces slow dissociation, while optical single-molecule methods generally require controlled imaging and substantial data processing. A 2026 study combined kinetic modeling with reversible single-molecule nanoswitch experiments and predicted that low-picomolar biomarkers could be quantified on approximately ten-minute timescales under realistic design conditions [38]. The conceptual advance is important: digital event counting can recover sensitivity while allowing lower-affinity, faster-resetting binders. Nevertheless, optical continuous monitoring still faces substantial challenges in miniaturized illumination, focus stability, background suppression, surface regeneration, and long-term operation in moving biological environments. Optical transduction is therefore strongest for ultrasensitive and multiplexed diagnostics, with continuous monitoring emerging but not yet comparably mature.

### 3.3. Field-Effect-Transistor and Graphene Transduction

Field-effect biosensors convert a change in surface charge or interfacial potential into the modulation of channel conductance. Their attraction is a direct, label-free electronic readout with the potential for rapid response, dense arrays, and semiconductor-scale manufacturing. Large-area organic transistors have reported single-molecule sensitivity [73], while aptamer-functionalized transistors demonstrated that binding-induced conformational changes can move charge within the electrostatically detectable region and thereby extend FET sensing to small molecules [23]. Graphene is especially attractive because its two-dimensional channel is entirely exposed to the sensing environment and supports high carrier mobility and straightforward surface functionalization.

Recent transistor-based platforms further illustrate the analytical sensitivity attainable through advanced electronic transduction. An aptameric field-effect transistor for cortisol sensing achieved a reported detection limit of 1 fM in a lab-on-chip configuration [74]. Organic electrochemical transistor (OECT) approaches have similarly extended dopamine biosensing from conventional enzymatic formats toward aptamer- and transistor-based architectures operating in the picomolar-to-femtomolar range [75]. These advances demonstrate the analytical leverage of emerging transistor architectures, while also reinforcing the central premise of this review: ultralow detection limits alone do not establish translational readiness without matrix robustness, calibration stability, device reproducibility, and intended-use validation.

Prominent demonstrations include the antibody-functionalized graphene detection of SARS-CoV-2 antigen in nasopharyngeal specimens [76] and an integrated platform containing more than 200 graphene sensing units, custom electronics, and machine-learning correction for device variability [77]. Multiplexed GFETs have differentiated SARS-CoV-2 and influenza targets [78], and internally referenced arrays have measured pancreatic-cancer-associated exosomes in patient plasma [79]. Clinical-matrix studies have since expanded to chemokines in nasal swabs [80], exosomal miRNA-196a for pancreatic ductal adenocarcinoma with an anti-fouling solution-gated transistor [81], colon-cancer-derived exosomes using a portable planar-gate platform [82], and adrenocorticotropic hormone measured by reduced-graphene-oxide FETs with comparison to clinical methods [83]. These studies indicate genuine movement from buffer demonstrations toward patient samples and integrated readout.

The central limitations, however, remain unresolved. In physiological ionic strength, Debye screening attenuates charge located more than a short distance from the transistor surface; antibodies and other large receptors can place the recognition event outside this region. Nonspecific adsorption, pH and ionic-strength changes, reference-gate instability, hysteresis, and wet packaging can produce signals comparable to target binding. Graphene synthesis, transfer, defects, contact resistance, and surface functionalization also create substantial device-to-device variability. Array redundancy, internal referencing, anti-fouling layers, and computational normalization can reduce these effects, but they do not substitute for blinded validation against accepted reference methods. Most recent clinical reports remain retrospective or use limited cohorts, and prospective external validation is rare. FET studies should therefore report not only detection limits but also device yield, batch variance, matrix-specific calibration, interference, drift, and diagnostic performance in representative populations.

### 3.4. Cross-Modality Performance Trade-Offs

No transduction mechanism is intrinsically superior; the optimal choice follows the use case. Electrochemical systems are generally best aligned with low-power wearable or minimally invasive monitoring because electrodes, microfluidics, and electronics can be co-integrated compactly. Optical digital assays are best suited to the centralized or near-patient measurement of very low-abundance proteins when instrument complexity is justified. Plasmonic and nanophotonic systems offer high sensitivity and multiplexing with potential portability, but packaging and spectral stability remain significant. FET and GFET platforms provide rapid, label-free electronic readout and scalable arrays, but their advantage depends on overcoming ionic screening, drift, and device variability in undiluted biological matrices. The principal advantages, limitations, best-fit applications, and current maturity of these transduction modalities are summarized in Table 1.

The trade-offs in Table 1 are therefore not fixed properties of each modality; current device engineering actively mitigates many of these limitations, although the effectiveness and maturity of those strategies vary substantially across platforms. Four performance dimensions should dominate comparison. First, matrix tolerance must be demonstrated in the intended sample rather than inferred from buffer. Second, temporal performance must include response and recovery times, drift, calibration stability, and sampling lag over the intended wear or assay period. Third, multiplexing should be evaluated as an analytical system: channel cross-talk, differential fouling, per-channel calibration, and the independent clinical value of each analyte matter more than channel count. Fourth, manufacturability requires device yield, batch-to-batch variance, shelf life, and reader compatibility. End-to-end design frameworks increasingly emphasize these coupled constraints [3], yet they remain inconsistently reported.

The practical hierarchy is therefore not electrochemical versus optical versus FET, but validated information versus impressive signal. A low detection limit obtained in buffer can establish physical feasibility; it cannot establish diagnostic accuracy or continuous-monitoring readiness. Conversely, a less sensitive sensor may be clinically superior if it is stable, factory-calibrated, low-power, reproducible, and validated against a reference method in the target population. Transduction should therefore be judged by the complete analytical chain—from molecular event and signal generation to calibration, biological sampling, data interpretation, and the decision the measurement is intended to support.

## 4. Device Architectures for Longitudinal Monitoring

**Figure 2:**
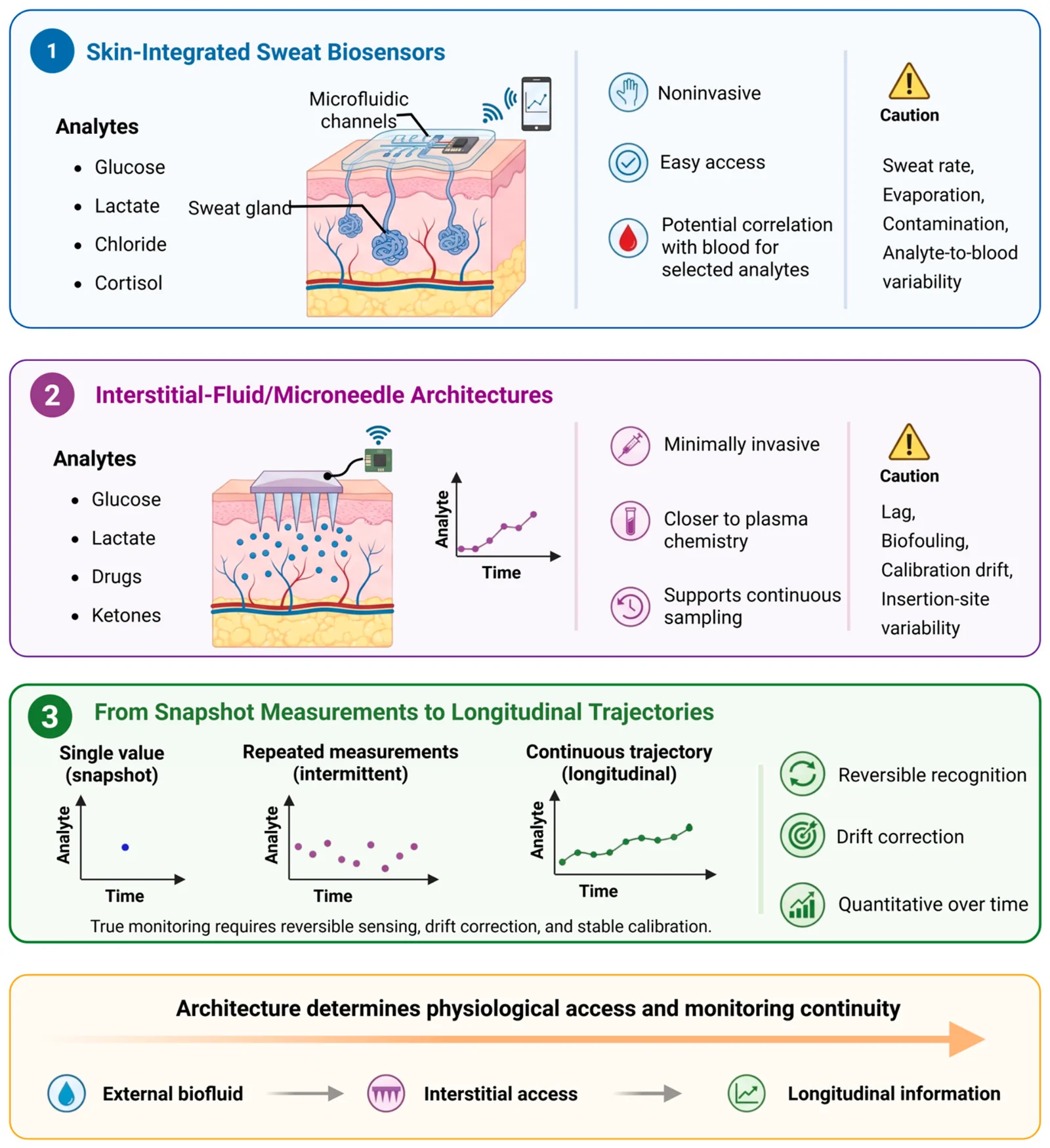
Device architectures for longitudinal monitoring. Sweat- and microneedle-based interstitial-fluid sensors enable snapshot, intermittent, and longitudinal measurements, with performance influenced by sampling variability, bio-fouling, physiological lag, and calibration drift. Created in BioRender. Roy, N. (2026) https://BioRender.com/ru9jmmn, accessed on 20 July 2026.

Transduction determines how a biosensor generates a signal; device architecture determines whether that signal becomes a clinically useful time series. This section specifically addresses architectures intended to generate repeated or longitudinal measurements; single-use and discrete diagnostic platforms are considered separately in Section 5. The central distinction is temporal. A snapshot assay measures an analyte at one time point, intermittent testing repeats discrete measurements, and continuous monitoring follows concentration changes with sufficient sampling frequency, reversibility, and stability to resolve a trajectory. The latter requires more than a sensitive transducer: the device must control access to the biological compartment, maintain contact and calibration, manage drift and fouling, and preserve data integrity during motion and changing environmental conditions. Architecture also determines the effective sampling volume, lag between physiology and measurement, analyte replenishment at the sensing surface, and whether reagents or waste products accumulate. These features can dominate performance even when two devices use the same receptor and transducer. This section therefore compares architectures by the information they can reliably produce, rather than by form factor alone. Figure 2 summarizes how sampling architecture influences biofluid access, physiological relevance, and the ability to generate longitudinal measurements.

### 4.1. Skin-Integrated Sweat Biosensors

Sweat is attractive because it can be sampled noninvasively and integrated with soft, skin-conformal microfluidics. Foundational systems established the main architectural elements now common in the field. Fully integrated electrochemical arrays combined multiplexed metabolite and electrolyte sensing with signal processing and wireless communication [56]. Soft microfluidic patches subsequently captured, routed, stored, and colorimetrically analyzed sweat in situ [84], while iontophoretic stimulation enabled autonomous sampling without exercise [85]. Battery-free near-field communication systems further coupled electrochemical, colorimetric, and volumetric measurements in a single epidermal platform [86]. Together, these studies shifted sweat sensing from isolated electrodes toward complete sample-to-data systems.

Architecture, however, cannot remove the biological limitations of the compartment. Sweat rate varies by body site, thermal load, hydration, and stimulation method; evaporation and skin contamination alter measured concentrations; and temporal relationships with blood are analyte-specific. Regional mapping showed substantial site- and rate-dependent variation, emphasizing that calibration cannot be transferred uncritically across locations or users [87]. Consequently, sweat should not be treated as a universal surrogate for plasma or interstitial fluid. For many analytes, the appropriate claim is local physiological monitoring unless a reproducible relationship with a clinical reference compartment has been established.

Recent devices increasingly address sampling control and actionability. A wearable electrochemical/biophysical system estimated whole-body sweat loss and sodium loss during occupational field use and delivered haptic feedback to the wearer [61]. An ultrasound-assisted platform later generated sweat under resting conditions and integrated uric-acid, pH, and potassium sensing, reducing dependence on exercise or pharmacological stimulation [62]. Protein and hormone monitoring have also become more integrated: a wireless C-reactive-protein patch combined iontophoresis, microfluidic reagent handling, calibration sensors, and electrochemical immunodetection [88]. A remote cystic-fibrosis study demonstrated the practical value of mailed microfluidic stickers and smartphone-supported sweat-chloride analysis, but also showed that remote exercise-induced measurements should not be presented as replacements for standardized diagnostic chloridometry [89]. Stressomic used controlled sweat induction and sequential microfluidics to profile cortisol, epinephrine, and norepinephrine during physical, psychological, and pharmacological challenges [90]. These studies move beyond detection toward controlled sampling and longitudinal use. Nevertheless, the architecture must report sweat induction method, local sweat rate, collection efficiency, residence time, evaporation control, and whether old and new samples mix within the channel. Without those data, a smooth wearable trace may reflect fluid transport and sensor memory as much as physiology. Clinical meaning therefore depends on analyte-specific validation, not merely on wearable integration.

### 4.2. Interstitial-Fluid and Microneedle Architectures

Interstitial fluid (ISF) offers closer exchange with plasma than sweat and is the biological compartment underlying commercial continuous glucose monitoring. Microneedles provide minimally invasive access while avoiding conventional venipuncture. Architectures range from hollow microneedles that extract fluid for downstream analysis [91] to solid or porous arrays that place electrochemical sensors directly within the dermis. Integrated systems have combined multiple sensing chemistries, wireless electronics, and on-body packaging for the simultaneous monitoring of glucose, lactate, and other biomarkers [57]. First-in-human drug-monitoring studies further established that microneedle electrochemistry can produce time-resolved pharmacokinetic profiles: phenoxymethylpenicillin measurements were compared to serum and microdialysis in healthy volunteers [59], and microneedle-enabled electrochemical aptamer sensors later tracked phenylalanine changes in human dermal ISF [92]. A pilot clinical trial of a wearable electrochemical aptamer patch extended this trajectory toward direct continuous drug-concentration measurement in people [36]. These studies also show why ISF cannot be treated as diluted blood. The measured lag and concentration relationship depend on molecular size, protein binding, local perfusion, tissue metabolism, and the sampling method; each analyte therefore requires its own compartment-validation study.

The main architectural challenges are sampling lag, insertion consistency, mechanical damage, tissue response, bio-fouling, and calibration drift. A sensor may perform well before insertion yet fail after the recognition layer is abraded or the microneedle is incompletely seated. Differential and redundant designs therefore matter as much as sensitivity. A differential microneedle glucose array used paired sensing and reference channels to reject common-mode interference [63], and a fully integrated multiplex array combined multiple microneedles with wearable electronics for fitness-related biomarkers [64]. More recent individually addressable arrays introduced redundant glucose-sensing channels and algorithm-triggered insulin release so that a single failed microneedle would not disable the system, although validation remained in rats [93].

Protein and therapeutic-monitoring architectures remain earlier in translation. A regenerable microneedle immunoassay generated repeated C-peptide measurements, but animal studies do not establish human continuous protein monitoring [60]. A zwitterionic protective interface enabled ongoing insulin measurement in diabetic rats [18]. Likewise, microneedle aptamer platforms have demonstrated continuous therapeutic-drug sensing [29], but duration, cohort size, calibration stability, and agreement with reference methods remain limited compared to glucose. The field should therefore distinguish short on-body feasibility, multi-hour trajectories, multi-day operation, and prospective clinical utility rather than grouping all under the term continuous monitoring.

### 4.3. From Repeated Measurements to Molecular Trajectories

A clinically meaningful molecular trajectory requires a chain of capabilities: reversible recognition, sufficiently rapid response and recovery, stable sampling access, drift correction, and calibration over the intended-use period. Electrochemical aptamer sensors established the principle by tracking circulating drugs in living animals [21,22]. Subsequent work showed that signal drift in whole blood arises from multiple time- and matrix-dependent processes [50], and that extended in vivo operation requires protective interfaces and stable reference strategies [35]. Xenonucleic-acid probes have now supported week-long, seconds-resolved drug measurements in vivo [37], while reversible single-molecule nanoswitches offer a complementary optical route for low-concentration monitoring [38]. The architecture must also preserve temporal fidelity: slow diffusion through membranes, long microfluidic residence times, receptor hysteresis, or digital filtering can smooth or delay the trace. Thus, nominal sampling frequency should not be confused with physiological time resolution. A device reading every minute does not provide one-minute biological resolution if fluid transport and binding kinetics impose a substantially longer response time.

These advances do not make every time series clinically actionable. Reporting should include sampling interval, response and recovery time, total monitoring duration, missing-data rate, calibration method, drift before and after correction, lag relative to the reference compartment, and the number and characteristics of human participants. The strongest benchmark remains continuous glucose monitoring, for which sensor performance has been linked to prospective clinical outcomes [65]. For emerging analytes, the translational goal is not merely to draw a curve but to demonstrate that the curve is accurate, reproducible, and useful for a defined decision.

### 4.4. Sensor-Integrated Organ-on-Chip and Microphysiological Systems

The preceding architectures generate measurements directly from patients or patient-derived specimens. Sensor-integrated organ-on-chip and microphysiological systems represent a distinct, tissue-facing application in which biosensors quantify dynamic biological states during disease modeling, toxicology, and drug evaluation. The broader engineering and translational development of organ-on-chip systems, including their integration with biosensing and translational workflows, has been reviewed previously [94,95]; the present section is limited to the measurement principles relevant to longitudinal biosensing, including temporal fidelity, calibration stability, biological perturbation, and reproducibility. Multisensor organ-on-chip platforms established that physical, biochemical, and optical measurements could be integrated with perfused microtissues for continual in situ assessment [96]. Early systems monitored glucose, lactate, oxygen, and other metabolites in three-dimensional spheroids [97], tracked mitochondrial dysfunction in liver-on-chip models through real-time metabolic measurements [98], and coupled electrochemical immunosensors with bioreactors to measure secreted biomarkers repeatedly [99]. These architectures changed the role of the biosensor from detecting a patient analyte to quantifying dynamic tissue state during disease modeling and drug exposure.

Recent platforms have improved both duration and functional breadth. Impedance spectroscopy has enabled the noninvasive monitoring of barrier formation and differentiation in organ chips [100]. Multianalyte electrochemical systems have followed oxygen, glucose, and lactate in three-dimensional cultures [101], and multifunctional gut–liver chips have combined barrier, oxygen, pH, metabolic, and imaging measurements under controlled oxygen conditions [102]. Newer optical and impedance architectures further illustrate the trend toward minimally disruptive, longitudinal readout: fiber-optic luminescence sensing tracked epithelial and endothelial barrier transport for nine days [103]; a miniaturized microoptical system monitored calcium dynamics in pancreatic islets in situ [104]; and a sensor-integrated gut-on-chip measured senescence-associated changes in barrier integrity over extended culture [105].

The translational endpoint for these systems is not clinical diagnosis but reproducible preclinical decision support. Sensor integration must not perturb the tissue or alter transport, oxygenation, or differentiation. Calibration must remain stable in warm, humid culture environments; sensor replacement and sterilization must be feasible; and results must agree with orthogonal biochemical, imaging, or histological measurements. Cross-laboratory reproducibility is especially important because biological model variability and sensor variability can otherwise be mistaken for drug effects. Device reports should therefore specify culture duration, sensor duty cycle, calibration frequency, drift, sterility controls, failed channels, and whether measurements were obtained continuously or at scheduled intervals. The most credible organ-on-chip platforms combine longitudinal sensing with explicit biological validation and standardized performance reporting. Across patient-facing and tissue-facing architectures, the same principle holds: device integration is valuable only when it converts a molecular signal into a reliable temporal record that supports a defined decision.

## 5. Clinical Applications and Translational Maturity

The clinical value of a biosensor depends on the decision it is intended to support, the temporal form of the measurement, and the consequences of an incorrect result. Infectious-disease testing generally requires a rapid diagnostic snapshot; cardiometabolic management may require an accurate trajectory over days; acute-care diagnostics require rapid quantitative measurements for triage; oncology screening requires high specificity in low-prevalence populations; and neurodegeneration increasingly uses blood biomarkers to guide confirmatory testing or treatment evaluation. Each domain is therefore considered according to its dominant measurement class, intended decision, most mature evidence cited, and principal translational gap. Selected centralized laboratory assays are included as translational comparators because they establish the validation and workflow standards that emerging integrated biosensors must ultimately match or exceed.

### 5.1. Infectious Diseases: Rapid Molecular Diagnosis

Infectious-disease biosensors are primarily designed to provide rapid diagnostic snapshots that identify a pathogen, strain, or clinically relevant sequence near the point of care. Their utility depends on whether the result is available soon enough to influence isolation, antimicrobial treatment, triage, or outbreak control. These systems therefore prioritize sample-to-answer operation, short turnaround time, internal controls, reagent stability, and decentralized robustness rather than prolonged longitudinal performance.

CRISPR-based diagnostics have substantially reduced the distance between programmable molecular recognition and portable testing. DETECTR combined preamplification with Cas12 recognition and lateral-flow readout for SARS-CoV-2, producing results in approximately 40 min and showing 95% positive agreement and 100% negative agreement with the U.S. Centers for Disease Control and Prevention RT-PCR assay in 78 respiratory samples [41]. Amplification-free Cas13 detection reduced workflow complexity through mobile-phone microscopy [42], while engineered Cas12a systems and multiplex microfluidic platforms expanded clinical validation and supported respiratory-virus and variant discrimination [43,44]. A Cas13-based SHERLOCK assay further demonstrated operation in authentic nasopharyngeal and throat specimens [106].

Recent studies have moved closer to intended-use deployment. A multi-guide Cas12a tuberculosis assay was evaluated across 603 clinical specimens, including prospectively collected tongue swabs [107]. During the 2025 mpox outbreak in Sierra Leone, the portable Mpox SHINE platform combined lyophilized reagents, ambient-temperature lysis, and automated fluorescence, showing complete concordance with qPCR in 56 field specimens [108]. Plasmonic and field-effect platforms provide complementary rapid-readout approaches [70,76,78], but their clinical evidence remains less extensive than that of nucleic-acid amplification workflows.

The most mature infectious-disease platforms have therefore reached authentic-specimen validation, multiplex testing, and limited field deployment. Their main remaining limitation is incomplete sample-to-answer integration. Extraction, amplification, contamination control, internal controls, waste handling, biosafety, reagent storage, and interpretation of weak or invalid signals remain separate engineering problems in many systems. Future studies should emphasize prospective accuracy, total time to actionable result, invalid-test rate, environmental robustness, and the successful use by the intended operator rather than analytical sensitivity alone.

### 5.2. Cardiometabolic Monitoring: Longitudinal Measurement and Closed-Loop Use

Cardiometabolic monitoring requires repeated or continuous measurements that accurately represent physiology over time. The resulting trajectory may support treatment adjustment, behavioral intervention, early warning, or closed-loop therapy. These systems must therefore demonstrate reversible sensing, stable calibration, characterized compartmental lag, acceptable missing-data rates, and performance over the intended wear period.

Continuous glucose monitoring remains the strongest translational benchmark. Its success reflects selective enzyme chemistry, characterized blood-to-interstitial-fluid kinetics, controlled mass transport, factory calibration, reproducible manufacture, integrated electronics, and treatment-relevant interpretation. Randomized trials have shown improved glycemic outcomes with intermittently scanned monitoring [65], improved time in range with closed-loop insulin delivery [109], and better glycemic control in adults with type 2 diabetes treated with basal insulin [110]. Real-world evidence has also expanded, although observational findings should not be interpreted as causal [111].

Expansion beyond glucose is technically credible but substantially less mature. Human studies have included a 28-day implantable near-infrared spectroscopy sensor for glucose, ketones, lactate, and ethanol [112]; a microneedle patch tracking beta-hydroxybutyrate in interstitial fluid [113]; first-in-human microneedle electrochemical monitoring of phenoxymethylpenicillin [59]; and a pilot electrochemical aptamer patch for continuous drug-concentration measurement [36]. Integrated microneedle arrays have also demonstrated multianalyte monitoring [57,63,64].

These studies establish increasingly sophisticated feasibility, but each added analyte requires its own evidence for blood-to-biofluid relationships, physiological lag, protein binding, local metabolism, interference, calibration transfer, and decision thresholds. A multianalyte device does not inherit the maturity of its glucose channel. For non-glucose platforms, the main gaps remain multi-day human accuracy, stable calibration, insertion-site and user variability, analyte-specific compartment validation, sensor replacement, and evidence that the trajectory improves treatment or outcomes.

### 5.3. Acute-Care Point-of-Care Diagnostics

Acute-care biosensors address a different problem: rapid quantitative snapshots for immediate triage, rule-in or rule-out decisions, or treatment initiation. Their performance should therefore be judged by diagnostic accuracy, agreement with central-laboratory comparators, turnaround time, invalid-test rate, operator usability, and integration into a validated clinical algorithm.

A point-of-care high-sensitivity cardiac-troponin-I assay was evaluated in 1102 emergency-department patients with acute chest discomfort and achieved discrimination comparable to established laboratory assays; an assay-specific 0/1 h algorithm safely ruled out myocardial infarction in a substantial proportion [114]. By contrast, a microfluidic digital NT-proBNP assay produced rapid results from a small whole-blood volume but was compared to a clinical method in only 15 serum samples [115]. The former supports multicenter clinical validation, whereas the latter remains primarily an analytical and workflow-feasibility study.

Commercial cartridge-based platforms further illustrate the clinical maturity attainable with single-use point-of-care sensing. The i-STAT Alinity system integrates disposable sensor cartridges with near-patient analysis and has demonstrated analytical performance across clinically relevant blood measurements. Such systems achieve clinical utility through controlled single-use formats, standardized calibration and quality control, and established workflows rather than prolonged in vivo sensor stability [116].

The principal translational requirement is reliable equivalence under time-critical conditions. Studies should therefore evaluate intended operators, emergency workflows, specimen-handling errors, indeterminate results, and the effect of faster measurement on triage, treatment, length of stay, or resource use. A rapid point-of-care result is clinically meaningful only when it preserves analytical quality and improves the decision pathway.

### 5.4. Oncology: Screening and Liquid-Biopsy Translation

Oncology makes the distinction between molecular detection and clinical diagnosis especially consequential. Blood-based assays can identify tumor-associated mutations, methylation patterns, fragmentation signatures, proteins, and extracellular vesicles at low abundance. Depending on intended use, these measurements may support screening, tissue-of-origin prediction, treatment selection, minimal residual disease assessment, or recurrence monitoring. Population screening imposes the greatest evidence burden because disease prevalence is low and false-positive results can lead to invasive investigations, serial imaging, anxiety, and cost.

CancerSEEK combined genomic alterations with circulating proteins across multiple surgically resectable cancers [117], while plasma methylome and fragmentation studies showed that cell-free DNA contains complementary information about tumor presence and tissue origin [118,119]. Device-scale platforms have included Exo-PROS for paired exosomal protein and microRNA signatures [120], as well as the graphene-based detection of pancreatic- and colorectal-cancer-associated extracellular vesicles or nucleic acids [79,81,82].

Many early studies used case–control designs comparing established cancers to selected healthy controls. Such designs demonstrate biological and analytical feasibility but often overestimate screening performance, where prevalence is lower, benign conditions create overlapping signals, and early-stage tumors release less circulating material. A high area under the receiver-operating-characteristic curve in an enriched cohort therefore does not establish useful positive predictive value in asymptomatic adults.

Prospective studies have begun to address this gap. DETECT-A screened 10,006 women using a multianalyte blood test followed by PET-CT and demonstrated that blood-based testing could enter an interventional diagnostic pathway while revealing the burden of confirmatory testing [121]. PATHFINDER returned multicancer early-detection results to 6621 evaluable participants and characterized downstream diagnostic resolution [122]. An independent validation of a targeted methylation-based multicancer test strengthened evidence for specificity and tissue-of-origin prediction, although the design remained case–control [123].

For colorectal cancer, ECLIPSE evaluated a cell-free-DNA test in an average-risk screening population and reported 83% sensitivity for cancer, approximately 90% specificity for advanced neoplasia, and 13% sensitivity for advanced precancerous lesions [124]. These data supported U.S. Food and Drug Administration approval of Shield in July 2024 for average-risk adults aged 45 years or older, with positive results requiring colonoscopy [125]. This is an important regulatory milestone, but it does not establish the replacement of established screening methods or validate broad multicancer screening.

Oncology has progressed from molecular proof of concept to prospective screening pathways and regulatory approval in a defined indication. Device-integrated extracellular-vesicle and graphene platforms remain earlier in translation. The principal gaps are stage-I and precancer sensitivity, prevalence-dependent predictive value, external validation, prespecified thresholds, diagnostic-resolution burden, and evidence that screening reduces late-stage incidence or mortality.

### 5.5. Neurodegeneration: Blood Biomarkers and Confirmatory Pathways

Neurodegeneration follows a different translational pattern. Biomarker validity has advanced rapidly, but measurements remain predominantly laboratory-based snapshots rather than wearable or continuous outputs. Blood biomarkers may be used to rule out pathology, identify patients for cerebrospinal-fluid or positron-emission-tomography confirmation, support treatment eligibility, or monitor response. Each use requires different thresholds and evidence.

Single-molecule digital immunoassays enabled the reliable measurement of dilute brain-derived proteins in plasma [2,66,67,68,69]. Plasma phosphorylated-tau species, especially p-tau217 and p-tau181, have shown the strong discrimination of Alzheimer disease pathology from other neurodegenerative disorders and have approached cerebrospinal-fluid or positron-emission-tomography reference performance in selected cohorts [126,127,128,129].

The evidence base has moved closer to intended workflows. A 2024 prospective study evaluated predefined blood-biomarker cutoffs in 1213 patients undergoing cognitive assessment in primary and secondary care and reported approximately 88–92% diagnostic accuracy [130]. A fully automated p-tau217 assay was subsequently evaluated across primary- and secondary-care cohorts using a two-cutoff strategy that retained an intermediate zone for confirmatory testing [131]. In 2025, the FDA cleared the Lumipulse G pTau217/beta-amyloid 1–42 plasma ratio and later the Elecsys pTau181 plasma assay for selected symptomatic populations [132,133]. These decisions support standardized laboratory workflows, not population screening or continuous neurological monitoring.

Population heterogeneity remains consequential. Longitudinal and diverse-cohort studies have shown that associations, discrimination, and optimal thresholds may vary with age, APOE genotype, race, and ethnicity [134,135]. The main unresolved issues are platform-dependent cutoffs, preanalytical variation, biological covariates, intermediate-result pathways, interlaboratory quality control, population generalizability, and evidence that blood testing improves treatment selection or patient outcomes. These assays should therefore be interpreted as components of a diagnostic pathway rather than stand-alone screening tools.

### 5.6. Cross-Domain Synthesis

The application areas have advanced through different translational pathways because they require different temporal outputs and tolerate different forms of uncertainty. Continuous glucose monitoring has the most mature longitudinal evidence base. Infectious-disease platforms provide the strongest examples of rapid decentralized molecular snapshots. Acute-care diagnostics show how point-of-care measurements must be embedded in validated triage algorithms. Oncology faces the highest burden for specificity and downstream diagnostic resolution, whereas neurodegeneration has advanced mainly through standardized laboratory assays and confirmatory pathways. These differences in measurement class, intended decision, evidence maturity, and principal translational gaps are summarized in Table 2.

Across these domains, translational maturity is determined by the depth of evidence surrounding the measurement rather than by the sophistication of the recognition chemistry or transducer. The next advances are most likely to arise from prospective intended-use studies, standardized preanalytics, calibration and quality-control systems, reproducible manufacture, and evidence that the result changes a clinical decision. A biosensor becomes consequential only when the complete pathway from the sampled compartment to interpretation and action is reliable.

Cardiometabolic sensing is most mature for longitudinal use because continuous glucose monitoring combines durable measurement, reproducible manufacture, workflow integration, and randomized clinical benefit, although this maturity remains largely confined to glucose [65,109,110,111]. Infectious-disease and acute-care platforms are further advanced for rapid snapshot decisions, with evidence extending to authentic-specimen testing, field deployment, and multicenter point-of-care validation [41,44,106,107,108,114]. Neurodegeneration has achieved prospective care-setting validation and regulatory clearance for selected plasma assays [130,131,132,133], while population-specific calibration, generalizability, and equitable implementation remain unresolved [134,135]. Oncology has reached prospective screening pathways and regulatory approval for a defined colorectal-cancer screening indication but continues to face challenges in early-stage sensitivity, false-positive resolution, and demonstrated outcome benefit [121,122,123,124,125].

## 6. Translation, Clinical Validation, and Data Integration

**Figure 3:**
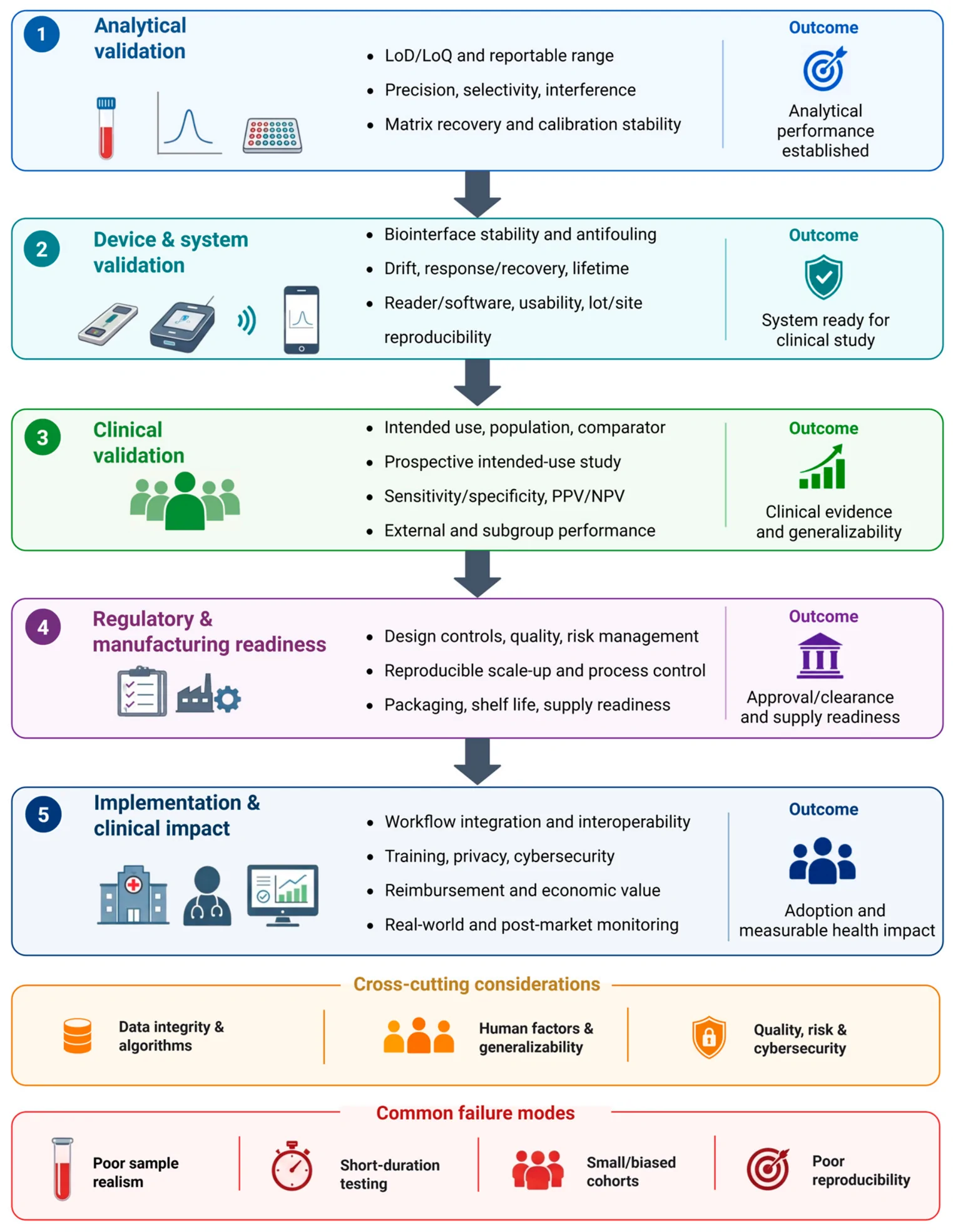
Translational pathway for diagnostic biosensors. The claim-matched, iterative pathway progresses from analytical and system validation through clinical validation, regulatory and manufacturing readiness, and real-world implementation, while highlighting cross-cutting requirements and common failure modes. Created in BioRender. Roy, N. (2026) https://BioRender.com/4xhv18i, accessed on 20 July 2026.

Translation requires demonstrating that the complete measurement system is fit for a defined use. That system includes the recognition element and biointerface, transducer, specimen-access method, calibration model, reader, software, operator workflow, manufacturing process, and decision pathway. Promising biosensors often fail when early testing omits intended-use stresses: complex matrices reduce selectivity, fouling and receptor degradation produce drift, sampling varies across users or sites, and hand-fabricated devices do not reproduce across lots [3,30,33,35,36,37,48,49,50,51,70,71,72,73,76,77,78,79,80,81,82,83,84,85,86,87,88,89,90,91,92,93]. The next experiment should therefore be determined by the claim being made rather than by the possibility of achieving a lower detection limit. Figure 3 summarizes the claim-matched and iterative pathway from analytical validation through clinical implementation, together with cross-cutting requirements and common translational failure modes.

### 6.1. Claim-Matched Analytical and Clinical Validation

Validation should begin with an intended-use statement defining the analyte or derived output, specimen, population, setting, operator, measurement frequency, decision threshold, comparator, and associated action. Analytical studies should then establish the characteristics relevant to that use. Quantitative assays generally require precision, reportable range, detection capability, interference testing, matrix equivalence, recovery, carryover where applicable, calibration stability, metrological traceability and measurement uncertainty where applicable, and reagent or sensor stability. CLSI and ISO frameworks provide established approaches for precision, interference, patient-sample comparison, detection capability, qualitative examination performance, stability, and laboratory quality [6,7,8,9,10,11,12], but the selected experiments must remain specific to the device architecture and intended claim.

Evidence requirements differ by measurement class. A qualitative infectious-disease test should be evaluated near the decision threshold and report invalid or indeterminate results in addition to sensitivity and specificity. A quantitative point-of-care assay requires agreement with an accepted laboratory comparator across the clinically relevant range, together with operator and environmental robustness. A screening test requires prospective evaluation in a population resembling the intended screening population because prevalence and disease spectrum strongly affect predictive value. Continuous or near-continuous monitoring additionally requires response and recovery time, drift before and after correction, calibration stability, physiological and transport lag, missing-data rate, wear duration, and performance across sensor replacements. Nominal sampling frequency alone does not establish temporal accuracy.

Clinical validation asks whether the complete system maintains performance in the intended population and workflow. Sensitivity and specificity should be accompanied by confidence intervals, prevalence-relevant predictive values, subgroup performance, participant flow, prespecified thresholds, and the handling of failed or indeterminate tests. FDA guidance addresses statistical reporting for binary diagnostic tests, while STARD provides a minimum reporting structure for diagnostic-accuracy studies [13,14]. Nonrepresentative patient selection and inconsistent reference standards can inflate apparent performance [15]. Small case–control cohorts can establish feasibility, but they should not be presented as intended-use validation.

A practical evidence sequence includes analytical proof of concept, intended-matrix performance, authentic specimens, retrospective clinical evaluation, prospective intended-use validation, workflow integration, reproducible manufacture, regulatory readiness, and demonstrated clinical or preclinical benefit. These stages are claim-dependent rather than a universal linear pathway: a rapid diagnostic assay, continuous monitor, and tissue-facing microphysiological sensor require different endpoints. Each publication should nevertheless limit its conclusions to the most mature evidence stage actually demonstrated.

### 6.2. Connected Readers, Data Integration, and Sensor-Plus-Algorithm Platforms

Portable readers, smartphones, and wireless electronics can reduce instrument burden, automate signal interpretation, and support remote monitoring. They also expand the validated system boundary. Smartphone microscopy has enabled amplification-free CRISPR detection, while wearable and outbreak platforms have used phone-based or wireless readout [42,61,84,85,86,87,88,89,90,108]. The relevant unit of validation is therefore the full path from specimen or sensor interface to the displayed and stored result. Illumination, camera characteristics, battery state, wireless interruption, operating-system changes, user interaction, and software version can alter performance even when the sensing chemistry is unchanged.

Human-factor engineering and software controls should be developed alongside the assay. IEC 62366-1 and IEC 62304 address usability engineering and medical-device software lifecycle processes [136,137], while remote measurements require fit-for-purpose verification, training, data protection, and risk management [138]. When results enter clinical systems, units, timestamps, calibration state, uncertainty, provenance, and missingness must remain attached to the measurement. HL7 FHIR can support interoperable exchange, but interoperability does not establish analytical or clinical validity [139]. Connected devices must also address confidentiality, integrity, availability, secure updates, and recovery throughout the product lifecycle [140].

Machine learning is most defensible when it interprets multiplexed signals, compensates for characterized device variation, or extracts information distributed across a trajectory. Graphene arrays and nanophotonic or digital platforms have coupled sensor outputs to multivariable models [72,77,115]. These models create an additional measurement layer whose inputs, preprocessing, parameters, thresholds, and update rules require validation. Development and test data should be separated by participant, site, and preferably device lot; random splitting of repeated measurements from the same participant can produce leakage. Evaluation should include calibration as well as discrimination, external-site performance, subgroup analysis, missing-data behavior, and sensitivity to sensor drift.

TRIPOD+AI, PROBAST+AI, STARD-AI, DECIDE-AI, and CONSORT-AI distinguish model development, risk of bias, diagnostic accuracy, live workflow testing, and clinical benefit [141,142,143,144,145]. Domain shift remains a practical concern because models may learn site, device, or workflow characteristics rather than the intended biological signal [146,147]. For modifiable AI-enabled devices, planned changes and their validation should be defined prospectively [148,149]. An algorithm can reduce characterized variability, but it cannot reliably compensate for biased recruitment, unstable chemistry, unmeasured drift, or an invalid reference standard.

### 6.3. Regulatory, Manufacturing, and Implementation Readiness

Regulatory requirements vary by jurisdiction, intended use, risk classification, and the claims made for the device. Regulatory readiness nevertheless begins with design controls, traceability, and risk management rather than the preparation of a final submission. ISO 13485 and ISO 14971 provide widely used frameworks for medical-device quality and risk management [150,151]. In the United States, the Quality Management System Regulation became effective on 2 February 2026 and incorporates ISO 13485:2016 by reference within 21 CFR Part 820 [152]. Wearable, microneedle, and implantable systems additionally require biological evaluation matched to the nature and duration of tissue contact [153]. Home-use and connected devices must account for lay users, variable environments, foreseeable misuse, maintenance, labeling, and lifecycle security [154,155].

Manufacturing evidence should address independent lots, critical-material specifications, process tolerances, receptor density, calibration transfer, device yield, packaging, shipping stress, shelf life, sterilization where applicable, and end-of-line quality controls. These factors often determine whether a laboratory prototype can be reproduced at scale. Late-stage studies should identify the number of devices fabricated and excluded, between-device variation, storage conditions, and whether testing spanned independent production runs.

Implementation introduces a separate evidence layer. A technically valid device may fail because it adds steps to an overloaded workflow, produces alerts without a response pathway, lacks reimbursement, or transfers work without clear benefit. Relevant outcomes include acceptability, adoption, feasibility, fidelity, cost, penetration, and sustainability [156]. The NASSS framework further emphasizes interactions among the condition, technology, value proposition, adopters, organization, wider system, and adaptation over time [157]. These considerations should be incorporated early because workflow fit, service requirements, and economic constraints can alter the optimal sensor architecture.

After deployment, complaint trends, calibration failures, software changes, cybersecurity events, subgroup performance, and real-world sensor drift should feed back into risk management, design controls, and post-market evaluation. Post-deployment evidence is particularly important for connected or algorithm-enabled systems whose performance may change with software updates, user behavior, operating environments, or shifts in the tested population.

## 7. Future Directions

The next phase of biosensor development should prioritize stable, interpretable, and decision-relevant measurements under intended-use conditions rather than further reductions in detection limits alone. Across recognition chemistries, transducers, diagnostic platforms, wearable systems, and tissue-facing models, five priorities emerge: durable biointerfaces, kinetically matched recognition, integrated sample-to-answer operation, sustained human monitoring, and validation-ready scale-up.

### 7.1. Durable Biointerfaces and Drift-Resilient Sensing

Durable operation in complex biological environments remains a central requirement. Anti-fouling coatings, chemically stable monolayers, protected recognition layers, regenerable interfaces, and differential reference channels should be treated as core device functions rather than late-stage corrections. Continuous systems also require referencing or recalibration strategies that distinguish physiological change from fouling, receptor degradation, temperature effects, and baseline drift. Week-long aptamer operation, protected in vivo interfaces, and mechanistic analyses of drift show that meaningful extension of sensor lifetime is achievable [30,35,37,48,50]. Progress should be assessed through retained accuracy, response and recovery, calibration stability, data completeness, and device-to-device reproducibility over the intended operating period rather than initial signal amplitude alone.

### 7.2. Recognition Elements Engineered for Kinetics and Physiological Conditions

Recognition elements should be engineered for the concentration range, sampling interval, and the matrix of the intended application rather than selected solely for equilibrium affinity. High-affinity binding can improve endpoint sensitivity but may slow dissociation and prevent a sensor from following decreasing concentrations. Aptamers, antibody switches, and synthetic affinity reagents should therefore be evaluated under realistic ionic strength, temperature, nuclease exposure, matrix competition, and surface-tethered conditions, with association and dissociation kinetics, switching amplitude, and chemical stability treated as primary variables. High-throughput switch conversion, single-molecule kinetic analysis, active-reset antibody systems, and nuclease-resistant xenonucleic-acid receptors provide complementary strategies [16,17,24,25,26,27,37,38]. The relevant endpoint is a receptor–interface combination that repeatedly tracks concentration changes within the required physiological range and temporal resolution.

### 7.3. Integrated Sample-to-Answer Molecular Diagnostics

For CRISPR and other programmable nucleic-acid platforms, molecular recognition is no longer the only major bottleneck. Translation requires the integration of lysis, extraction, sufficient sensitivity with or without amplification, contamination control, reagent storage, internal controls, result interpretation, and waste containment within a closed workflow. Systems should be evaluated by total sample-to-result time, operator steps, invalid-test rate, storage stability, environmental tolerance, and performance outside specialist laboratories. Multiplex clinical assays and outbreak deployment demonstrate that portable CRISPR diagnostics are feasible [42,43,44,106,107,108], but broader adoption will depend on cartridge-level integration, automated quality control, and reproducible operation by intended users rather than the continued optimization of isolated cleavage reactions.

### 7.4. Multi-Day Human Monitoring and Decision-Relevant Trajectories

Wearable, microneedle, and interstitial-fluid systems must progress from short demonstrations to multi-day human evaluation. Studies should characterize insertion consistency, physiological and transport lag, site variability, local tissue response, bio-fouling, calibration transfer, missing data, device replacement, and adherence over clinically relevant durations. Evidence spanning early human feasibility and longer animal studies—including aptamer patches, microneedle platforms, ketone monitoring, and week-long in vivo drug sensing—indicates a credible path beyond glucose [18,36,37,57,63,92,93,113].

A longer trace alone is insufficient. The trajectory must agree with an accepted reference method across rising and falling concentrations and support a defined action, such as dose adjustment, early-warning detection, or closed-loop intervention. Continuous glucose monitoring remains the benchmark for connecting longitudinal measurement accuracy with treatment benefit [109,110,111].

### 7.5. Validation-Ready Platforms and Reproducible Scale-Up

Future platforms should be designed around an explicit intended use, comparator, operator, operating duration, manufacturing process, and decision pathway from the outset. Tissue-facing microphysiological systems require stable calibration, standardized biological challenges, orthogonal validation, and cross-laboratory reproducibility before sensor-derived trajectories can support preclinical decisions [96,100,101,102,103,104,105]. Sensor-plus-algorithm systems require participant-level separation of development and test data, external-site and device-lot evaluation, calibration assessment, subgroup analysis, and monitoring for drift and domain shift [141,142,143,144,145,146,147,148,149]. Longitudinal outputs should retain provenance, uncertainty, calibration state, and missingness metadata.

Scale-up should be demonstrated rather than inferred from individual prototypes. Future studies should include independent production lots, predefined acceptance criteria, device yield, calibration transfer, storage stability, intended-user performance, and monitoring after deployment. Quality systems, biocompatibility, packaging, cybersecurity, maintainability, workflow fit, and economic feasibility should be incorporated early enough to influence design rather than addressed only after technical development [150,151,152,153,154,155,156,157].

## 8. Conclusions

Biosensors are evolving from isolated analytical detectors into integrated measurement systems in which molecular recognition, biointerface engineering, transduction, sampling, calibration, data processing, and workflow are co-designed. Enzymes remain the most mature basis for catalytic continuous sensing; aptamers and resettable antibody systems are expanding reversible molecular monitoring; and CRISPR provides programmable recognition for rapid nucleic-acid diagnostics. Electrochemical platforms are best suited to low-power wearable and minimally invasive monitoring, whereas optical and digital systems provide exceptional sensitivity for discrete protein and nucleic-acid assays. Field-effect platforms offer rapid, label-free electronic readout but remain limited by ionic screening, drift, wet-interface instability, and device variability. Across all modalities, reliable performance in complex biological environments depends as much on the biointerface, sampling architecture, and calibration strategy as on the receptor or transducer.

The distinction among diagnostic snapshots, repeated discrete measurements, and genuine molecular trajectories is fundamental. Infectious-disease and acute-care platforms primarily require rapid, accurate results within complete sample-to-answer workflows. Cardiometabolic sensing has progressed furthest toward clinically actionable trajectories, with continuous glucose monitoring combining durable chemistry, reproducible manufacturing, workflow integration, and demonstrated clinical benefit. Oncology and neurodegeneration have advanced through ultrasensitive blood-based measurements, but most remain discrete laboratory assays whose value depends on representative populations, predefined thresholds, confirmatory pathways, and evidence beyond analytical discrimination. Sensor-integrated microphysiological systems extend these principles to longitudinal tissue assessment, where calibration stability, biological validation, and reproducibility determine their value for preclinical decisions.

The technologies closest to meaningful translation nevertheless occupy distinct positions. Continuous glucose monitoring represents the benchmark for longitudinal sensing because analytical performance, multi-day stability, reproducible manufacture, workflow integration, and clinical benefit have all been demonstrated. Cartridge-based acute-care and point-of-care systems likewise show that single-use biosensors can achieve clinical maturity without prolonged sensor operation, although new platforms must establish equivalence to accepted laboratory methods and demonstrate workflow benefit. CRISPR-based infectious-disease diagnostics have progressed to authentic-specimen testing, multiplexing, and limited field deployment but still require more complete sample-to-answer integration and prospective intended-user validation. Blood-based oncology and neurodegeneration assays have reached prospective validation and, for selected indications, regulatory clearance, but broad screening or stand-alone diagnostic claims require stronger evidence for generalizability, downstream clinical resolution, and patient benefit. Non-glucose wearable, microneedle, aptamer, and emerging transistor-based platforms remain promising but generally require longer human studies, stable calibration across devices and lots, reference-method agreement, and prospective evidence that their measurements improve decisions or outcomes.

These distinctions also define claims that should presently be avoided: a low detection limit in buffer should not be equated with clinical readiness; a short or repeatedly sampled trace should not be described as durable continuous monitoring; high discrimination in an enriched case–control cohort should not be interpreted as screening utility; single-site or small-cohort performance should not imply population generalizability; and the performance of individually fabricated prototypes should not be treated as evidence of manufacturing readiness. The central translational requirement is therefore claim-matched evidence that the complete measurement system performs reliably in its intended matrix, population, duration, workflow, and decision context.

## Figures and Tables

**Table 1:** Practical comparison of biosensor transduction mechanisms.

Modality	Main Advantage	Key Limitation	Representative Mitigation Strategies	Best-Fit Use and Maturity
Electrochemical [1,56,57,58,59,60,61,62,63,64,65]	Low power; inexpensive; easily miniaturized; compatible with wearables and microneedles	Fouling, reference drift, calibration, mediator/enzyme degradation, device variability	Anti-fouling coatings and selective membranes; reference/differential channels; controlled mass transport; calibration and drift correction	Wearable/minimally invasive continuous monitoring and disposable point-of-care assays; commercial for glucose, emerging for other analytes
Optical/fluorescence [2,66,67,68,69]	High sensitivity; flexible labels; strong spatial and spectral multiplexing	Optical components, background fluorescence, photobleaching, sample burden	Ratiometric or reference signals; microfluidic background control; improved labels and optical filtering; automated calibration	Single-use or instrumented discrete protein and nucleic-acid assays; clinically established in laboratory workflows
Digital/single-molecule optical [2,38,66,67,68,69]	Molecular counting; exceptionally low detection limits	Partitioning, imaging, data processing, cost, limited portability	Molecular partitioning and counting; pre-equilibrium readout; multiplexed bead formats; kinetic event analysis	Centralized instrumented discrete assays for low-abundance protein biomarkers; commercial for selected applications
Plasmonic/nanophotonic [54,70,71,72]	Rapid, label-free surface sensing; optical enhancement; multiplex potential	Temperature sensitivity, fouling, fabrication tolerances, spectral instability	Anti-fouling interfaces; temperature and spectral referencing; nanostructure optimization; internal controls	Portable or laboratory-based discrete assays for pathogens, proteins, and exosomes; laboratory to early clinical feasibility
FET/GFET [23,55,73,76,77,78,79,80,81,82,83]	Rapid, label-free electronic readout; compact arrays; scalable fabrication potential	Debye screening, gate drift, hysteresis, wet packaging, device-to-device variability	Short or conformational receptors; anti-fouling layers; reference gates; array redundancy; computational normalization	Portable or disposable discrete sensing of ions, pathogens, proteins, and exosomes; analytical to retrospective clinical validation

**Table 2:** Cross-domain comparison of biosensor applications and translational maturity.

Domain	Representative Format and Measurement Class	Intended Decision	Most Mature Evidence Cited	Principal Remaining Gap
Infectious diseases [41,44,106,107,108]	Single-use/portable point-of-care; rapid diagnostic snapshot	Pathogen identification, treatment, isolation, or outbreak control	Authentic-specimen validation, multiplex testing, and limited field deployment	Closed sample-to-answer operation, intended-user usability, invalid-test control, and decentralized quality assurance
Cardiometabolic monitoring [36,57,59,65,109,110,111,112,113]	Wearable/minimally invasive; continuous or near-continuous trajectory	Treatment adjustment, early warning, or closed-loop intervention	Randomized clinical benefit, closed-loop use, and broad implementation for glucose	Durable calibration, compartment correlation, multi-day human accuracy, and clinical utility for non-glucose analytes
Acute-care diagnostics [114,115,116]	Single-use cartridge/point-of-care; rapid quantitative snapshot	Emergency triage and rule-in or rule-out decisions	Multicenter clinical validation for point-of-care troponin	Demonstrated workflow and outcome benefit, operator robustness, and central-laboratory equivalence
Oncology [121,122,123,124,125]	Laboratory or device-integrated; screening/diagnostic snapshot or serial discrete testing	Early detection, localization, diagnosis, or recurrence assessment	Prospective screening pathways and FDA-approved blood-based colorectal-cancer screening	Early-stage and precancer sensitivity, false-positive resolution, external validation, and mortality benefit
Neurodegeneration [130,131,132,133,134,135]	Centralized laboratory assay; diagnostic snapshot	Rule-out, triage to confirmatory testing, or treatment evaluation	Prospective care-setting validation and FDA-cleared plasma assays	Standardized cutoffs, intermediate-result pathways, population generalizability, and demonstrated clinical benefit

## Data Availability

No new data were created or analyzed in this study. Data sharing is not applicable to this article.

## Abbreviations

The following abbreviations are used in this manuscript:

Abbreviation	Definition
AI	Artificial intelligence
APOE	Apolipoprotein E
Cas12	CRISPR-associated protein 12
Cas12a	CRISPR-associated protein 12a
Cas13	CRISPR-associated protein 13
CFR	Code of Federal Regulations
CGM	Continuous glucose monitoring
CLSI	Clinical and Laboratory Standards Institute
CONSORT-AI	Consolidated Standards of Reporting Trials–Artificial Intelligence
COVID-19	Coronavirus disease 2019
CRC	Colorectal cancer
CRISPR	Clustered regularly interspaced short palindromic repeats
crRNA	CRISPR RNA
DECIDE-AI	Developmental and Exploratory Clinical Investigations of Decision-support systems driven by Artificial Intelligence
DETECTR	DNA Endonuclease-Targeted CRISPR Trans Reporter
DigitISA	Digital immunosensor assay
DNA	Deoxyribonucleic acid
DNase	Deoxyribonuclease
EAB	Electrochemical aptamer-based
ELISA	Enzyme-linked immunosorbent assay
Exo-PROS	Exosome protein–microRNA one-stop biosensor
FDA	U.S. Food and Drug Administration
FET	Field-effect transistor
FHIR	Fast Healthcare Interoperability Resources
GFET	Graphene field-effect transistor
HABS-HD	Health and Aging Brain Study–Health Disparities
HbA1c	Glycated hemoglobin A1c
HL7	Health Level Seven International
IEC	International Electrotechnical Commission
IMDRF	International Medical Device Regulators Forum
ISF	Interstitial fluid
ISO	International Organization for Standardization
MCED	Multicancer early detection
miRNA	MicroRNA
NASSS	Nonadoption, abandonment, scale-up, spread, and sustainability
NT-proBNP	N-terminal pro–B-type natriuretic peptide
PET-CT	Positron-emission tomography–computed tomography
POC	Point of care
PROBAST+AI	Prediction model Risk of Bias ASsessment Tool + Artificial Intelligence
p-tau181	Tau phosphorylated at threonine 181
p-tau217	Tau phosphorylated at threonine 217
qPCR	Quantitative polymerase chain reaction
RNA	Ribonucleic acid
RT-PCR	Reverse-transcription polymerase chain reaction
S100B	S100 calcium-binding protein B
SARS-CoV-2	Severe acute respiratory syndrome coronavirus 2
SELEX	Systematic evolution of ligands by exponential enrichment
SHERLOCK	Specific High-sensitivity Enzymatic Reporter unLOCKing
SHINE	Streamlined Highlighting of Infections to Navigate Epidemics
STARD	Standards for Reporting Diagnostic-Accuracy Studies
STARD-AI	Standards for Reporting Diagnostic-Accuracy Studies–Artificial Intelligence
TRIPOD+AI	Transparent Reporting of a Multivariable Prediction Model for Individual Prognosis Or Diagnosis + Artificial Intelligence
